# Odontogenic Cutaneous Sinus Tracts: When Dental Pathology Makes Its Mark on the Skin

**DOI:** 10.7759/cureus.78355

**Published:** 2025-02-01

**Authors:** Akram Belmehdi, Soukayna Bahbah, Karima El Harti

**Affiliations:** 1 Oral Surgery, Faculty of Dental Medicine, Mohammed V University, Rabat, MAR

**Keywords:** odontogenic infection, odontogenic sinus tract, skin lesion, surgical management, misdiagnosis

## Abstract

Odontogenic cutaneous sinus tracts are relatively rare. They consist of a sinuous channel or cord-like tissue extending from a dental infection site to the face or neck. This channel follows the path of least resistance to externalize, with its localization influenced by factors such as the proximity of the dental apex to the cortical bone and muscle attachments, root length, and the morphology of the affected jaw.

Various etiologies can contribute to the development of these fistulas; however, chronic periapical abscesses caused by pulp necrosis due to dental caries represent the most common origin. These fistulas are frequently misdiagnosed as skin lesions due to the absence of overt dental symptoms. This delays appropriate treatment for these benign lesions and increases the risk of complications. Through a clinical case, we will explore the diagnostic challenges and discuss the therapeutic considerations specific to cutaneous sinus tracts of dental origin.

## Introduction

The odontogenic cutaneous sinus tract (OCST) is a pathological channel that forms between an infected tooth and the external skin surface, most often appearing on the face or neck. In medical and dental literature, the terms "fistulas" and "sinus tracts" are frequently used interchangeably to describe this condition. Although OCST is relatively uncommon, it holds substantial clinical importance due to the diagnostic and therapeutic challenges it presents [1,2].

This condition is predominantly associated with chronic periapical infections, such as periapical abscesses, or pulpal necrosis with periapical pathosis. However, its etiology may also include trauma, complications from dental implants, salivary gland lesions, neoplasms, infected cysts, osteomyelitis, tuberculosis, or fungal infections. The diagnostic complexity of OCST often arises from its ability to mimic other pathologies, such as granulomatous diseases, basal or squamous cell carcinoma, salivary gland and duct fistulas, furuncles, or actinomycosis [3,4].

Misdiagnosis remains a significant concern, with approximately half of affected patients undergoing repeated topical treatments, surgical procedures, or multiple rounds of antibiotics. This approach increases the risk of bacterial resistance and recurrence. Despite advancements in diagnostic techniques and treatment modalities, mismanaged cases of sinus tracts of dental origin are still reported in the literature [5,6].

Effective management requires identifying and eliminating the source of the infection to sever the communication between the infected site and the external skin. While non-surgical root canal treatment is often effective, some cases necessitate surgical-endodontic therapy to ensure complete resolution and prevent recurrence.

The aim of this article is to highlight the diagnostic challenges of odontogenic cutaneous sinus tracts and emphasize the importance of early recognition and appropriate dental management to prevent misdiagnosis and unnecessary treatments.

## Case presentation

A 57-year-old diabetic patient with a glycated hemoglobin level of 7.5% presented in the department of oral surgery of our institution with a cutaneous nodule on the mandibular right side, persisting for two years. The lesion exhibited intermittent swelling and occasionally discharged through a small opening. The patient reported consulting a general practitioner who had prescribed a dermatological cream for local application to the lesion, but it yielded no results.

**Figure 1:**
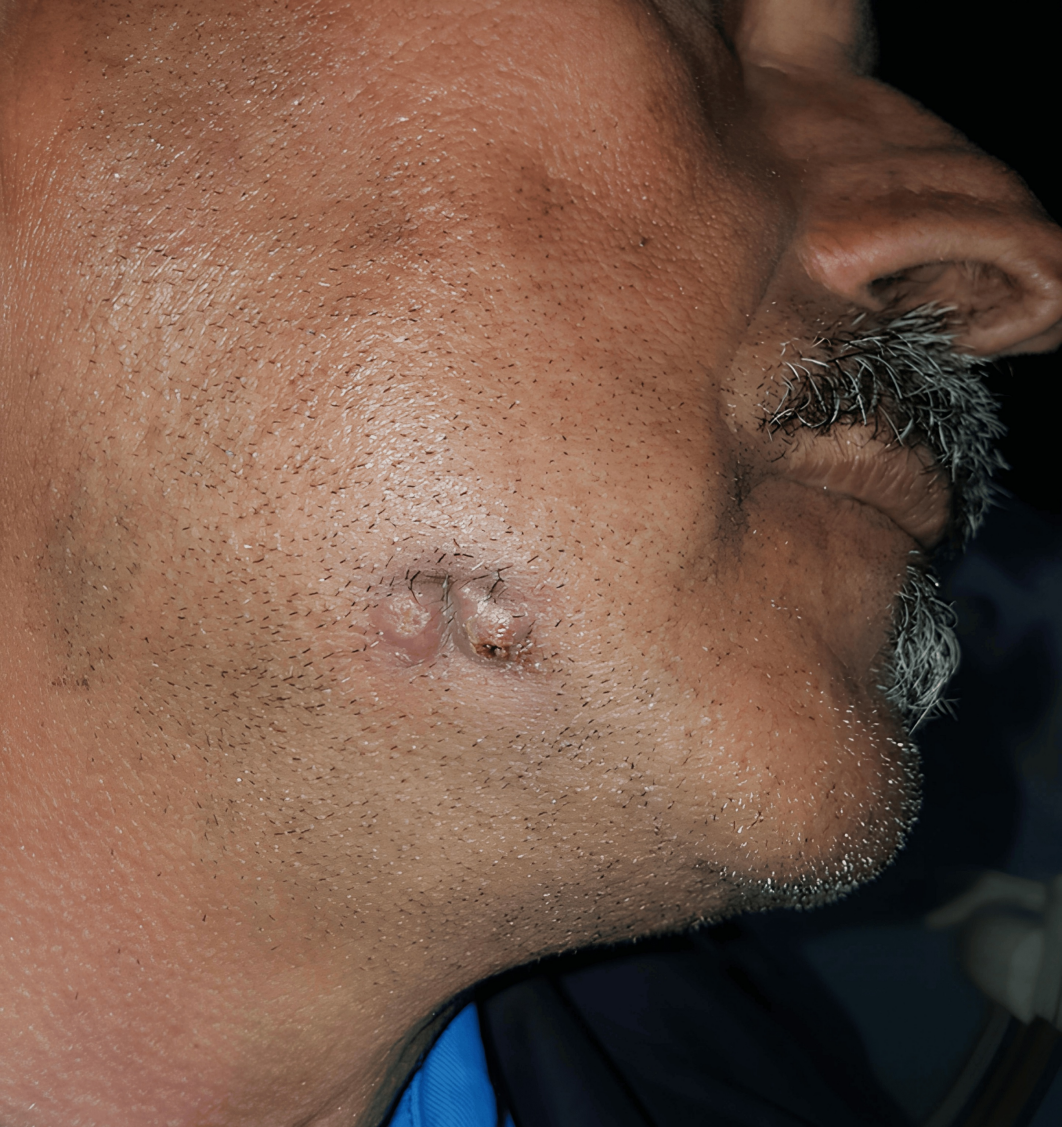
Extraoral view of the skin lesion showed a small bi-lobed masses

Extraoral examination revealed two small bi-lobed masses, each approximately 5 mm in diameter, resembling papules. One papule displayed a crust. Palpation was painless, and the overlying skin appeared slightly taut (Figure 1).

**Figure 2:**
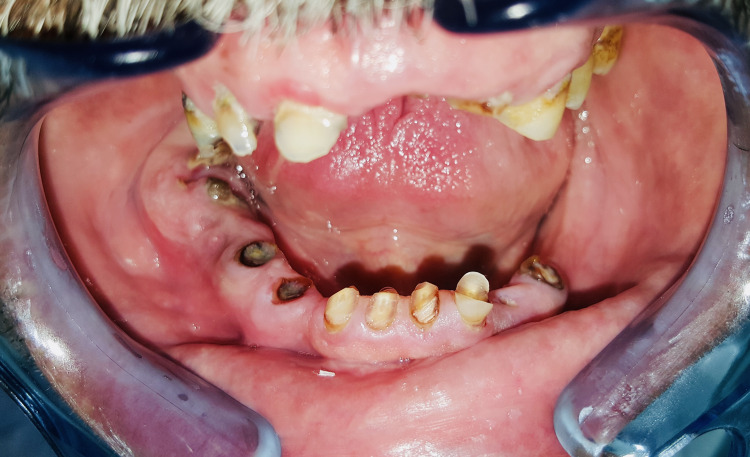
Intraoral view showed a periodontal inflammation and a palpable cord corresponding to decayed Teeth 46 and 47

Intraoral examination revealed poor oral hygiene, periodontal inflammation, and a palpable cord corresponding to the roots of remaining Teeth 46 and 47 (Figure 2).

**Figure 3:**
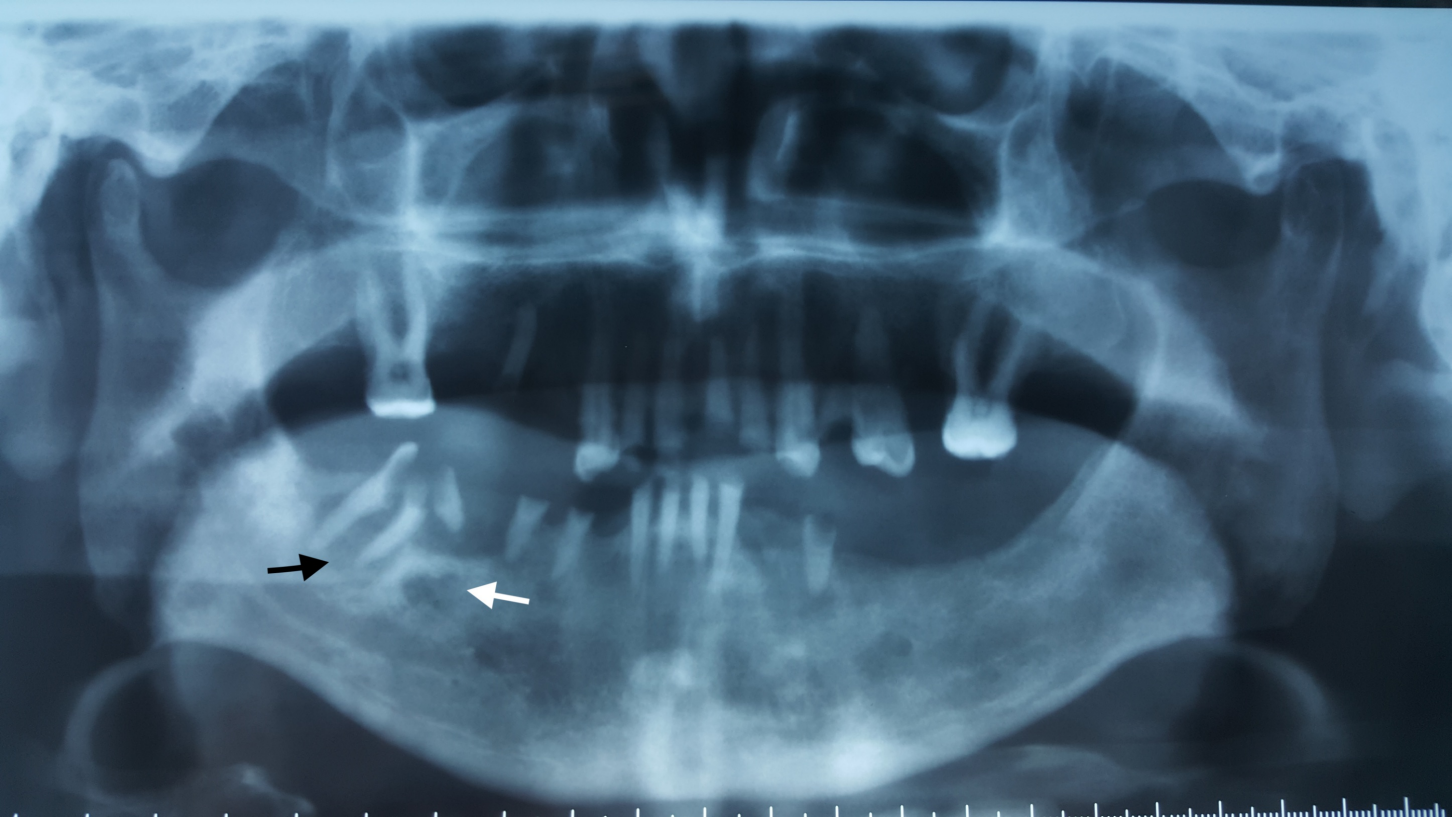
Orthopantomogram revealed two radiolucent lesions associated with the roots of 46 (white arrow) and 47 (black arrow)

A panoramic radiograph revealed two radiolucent areas associated with the roots of these teeth. Based on these findings, the initial diagnosis suggested a cutaneous fistula originating from the periapical lesion of Tooth 47 (Figure 3).

Due to the similarity of this cutaneous fistula to facial skin lesions observed in systemic pathologies, syphilis and HIV serology tests were prescribed to rule out other etiologies.

**Figure 4:**
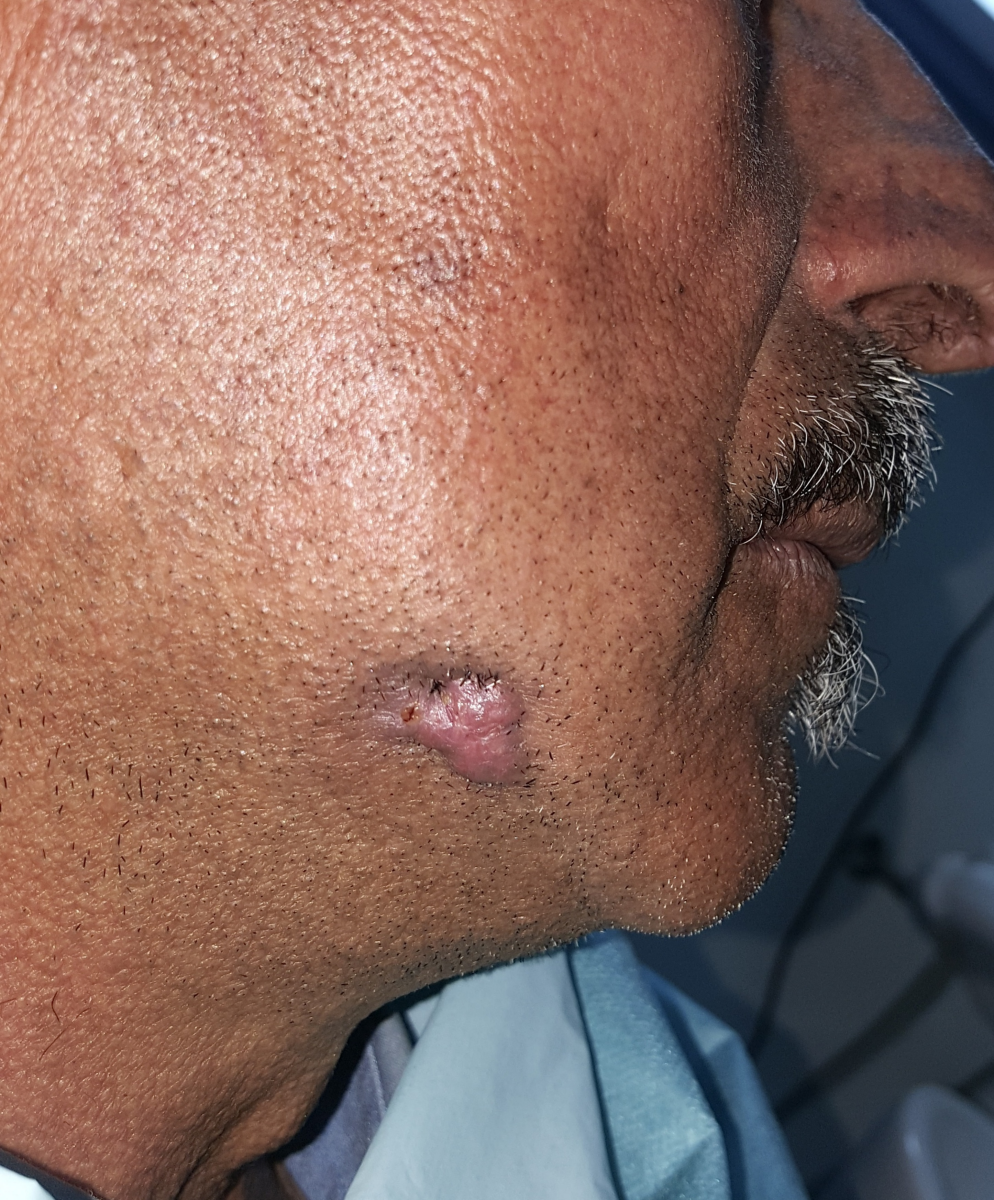
The skin lesion two months after the first consultation: A shape change

The patient was lost to follow-up for two months and returned with a cutaneous lesion now presenting as a single nodule approximately 1.5 cm in size, featuring a lateral orifice discharging serous fluid (Figure 4).

**Figure 5:**
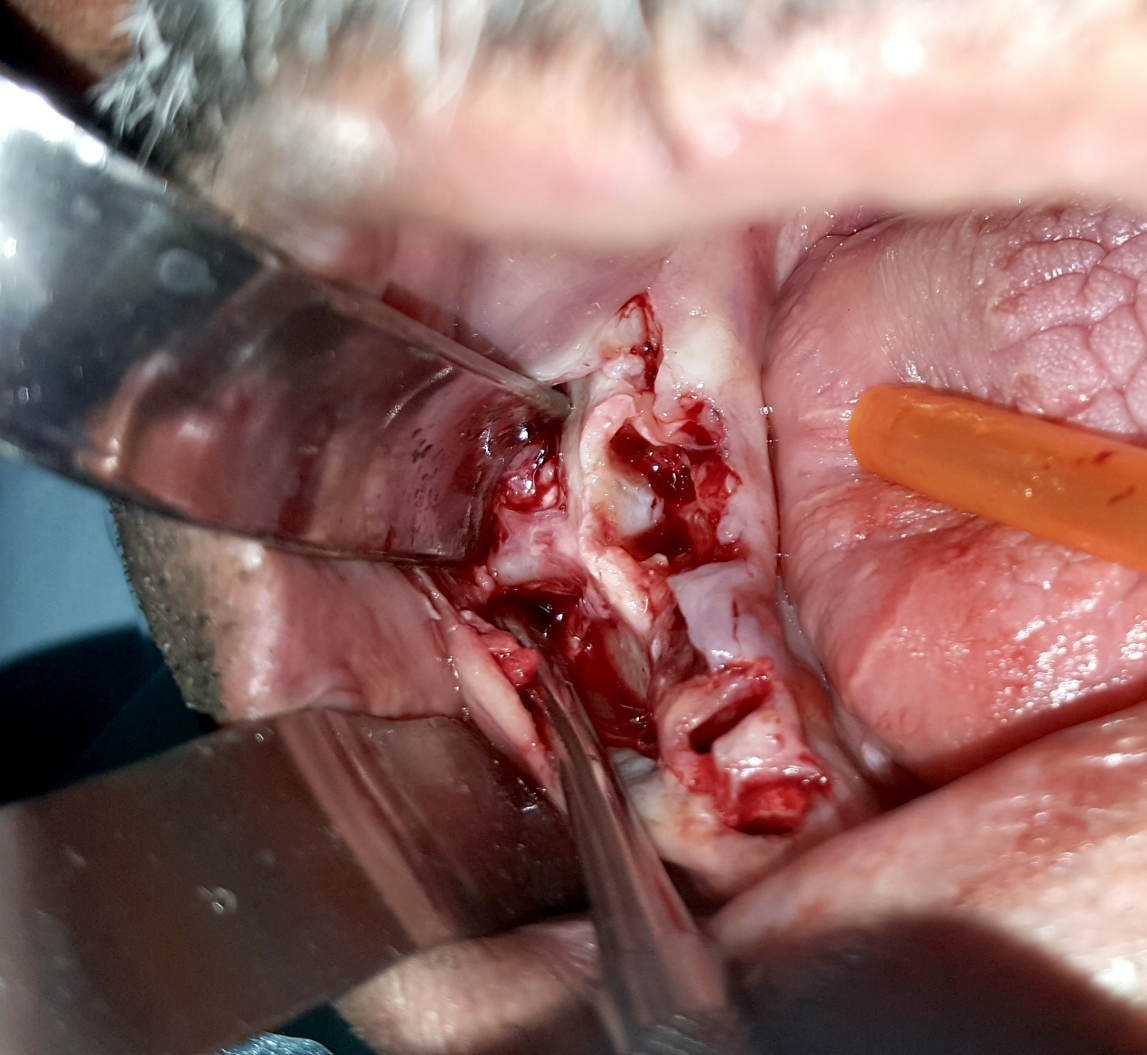
Peroperative view of the cord-like tract attached to the buccal cortical bone of Tooth 47

Serological tests were negative. Under local anesthesia (lidocaine 2% with epinephrine 1:100,000 i), Teeth 46 and 47 were extracted. Elevation of a mucoperiosteal flap revealed a fistulous tract attached to the buccal cortical bone of Tooth 47 (Figure 5).

**Figure 6:**
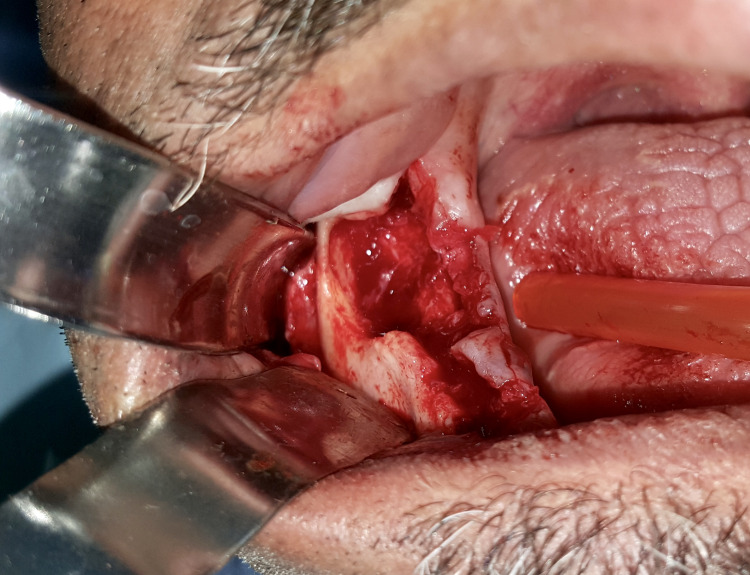
Detachment and dissection of the cord-like tract from its alveolar origin

The cord-like was meticulously dissected and excised, followed by thorough curettage of the periapical lesions (Figure 6). The prescribed medication included a paracetamol-based analgesic (one 500 mg tablet, three times a day as needed for pain) and a chlorhexidine-based antiseptic mouthwash, to be used for rinsing three times a day for 10 days.

**Figure 7:**
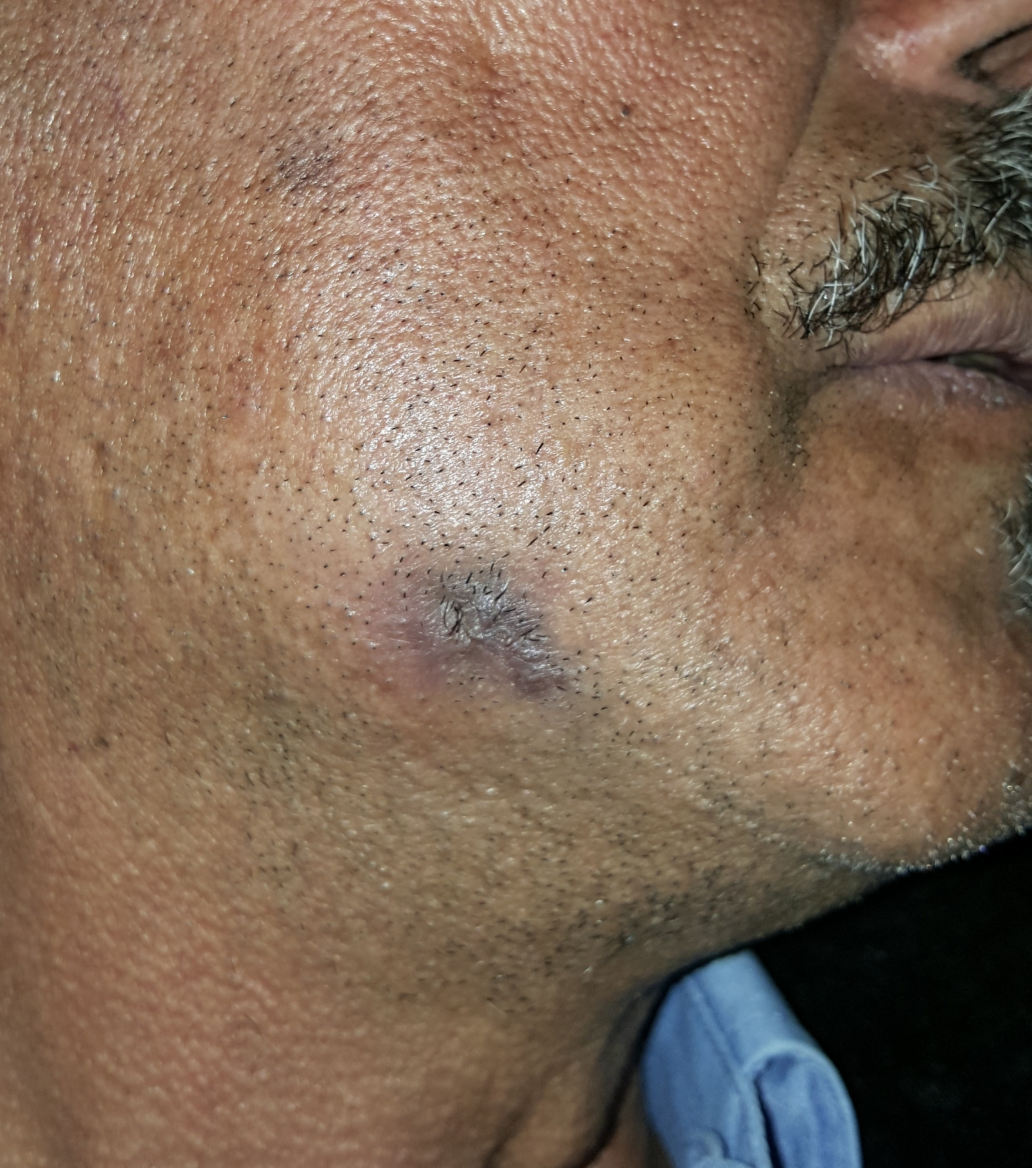
Clinical appearance of the cutaneous sinus tract after 10 days. Relaxation of the skin and flattening of the mass

At the three-week follow-up, the cutaneous wound demonstrated significant healing, characterized by relaxation of the skin and flattening of the mass, leaving a residual brownish macule (Figure 7).

**Figure 8:**
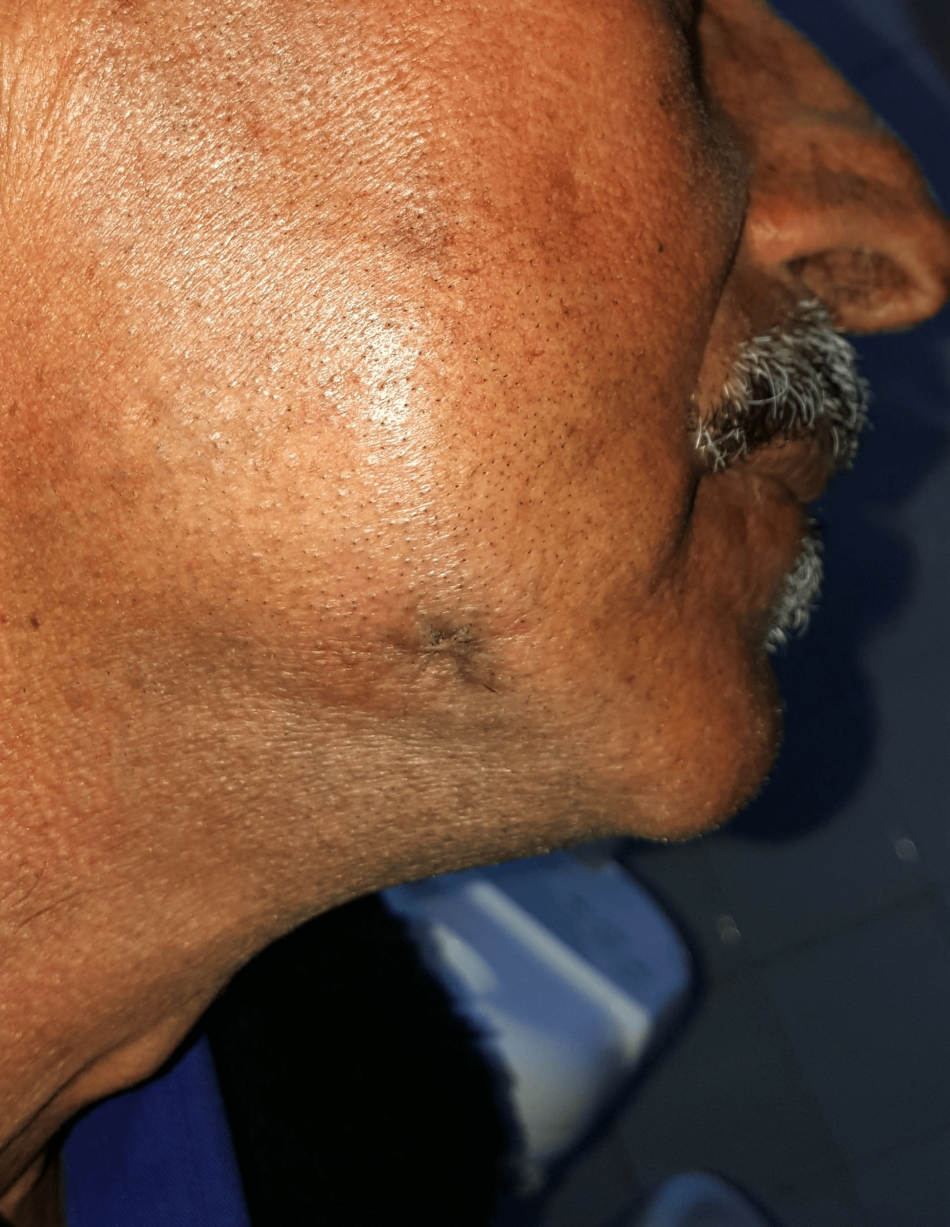
Healing of the skin lesion at two months with improvement of the aesthetic appearance

By the two-month follow-up, approximately 50% reduction in the size of the macule was observed with an enhanced aesthetic appearance of the previous lesion (Figure 8).

## Discussion

Chronic odontogenic infections typically manifest as intraoral draining sinuses. However, in rarer cases, these infections may progress and drain externally through the facial skin, resulting in an odontogenic cutaneous fistula or sinus [7].

Diagnosing odontogenic cutaneous fistulas can be particularly challenging for clinicians. It is estimated that nearly half of these cases (50%) undergo dermatological surgery or extended courses of antibiotics before the underlying cause is accurately identified [8]. This patient, like others, had used a cream prescribed by the general practitioner, without recognizing the potential dental origin of the fistula.

Studies concentrating on the prevalence of sinus tracts have demonstrated the relatively infrequent manifestation of their cutaneous expression [9].

Slutzky-Goldberg et al. in his cohort study involving an investigation of 108 odontogenic sinus tracts, observed only one occurrence of a cutaneous sinus tract [10]. Conversely, in the study conducted by Gupta and Hasselgren, which included 29 cases of odontogenic sinus tracts, all manifestations exhibited intraoral openings [11].

In a study conducted by Miri et al. in Iran, involving 1527 patients with a personal history of endodontic treatment, a frequency of 9.9% of sinus tracts without any cutaneous opening was observed [12]. Another study, reported by Sadeghi and Dibaei in 2011, examined a very similar population with the same history and under canalicular therapy, finding a prevalence of 14.7% of sinus tracts without an extraoral expression in 728 patients [13].

In the comprehensive research by Guevara-Gutierrez et al. [14]. it was observed that odontogenic cutaneous fistulas most commonly appeared at the mandibular angle (36%), followed by the chin (28%), and then the cheeks (24%). In virtually all cases (99%), the oral cutaneous fistula (OCF) was found next to the tooth responsible for the infection. The study also noted rarer locations for such fistulas that practitioners should be mindful of when examining facial skin lesions: these include the inner corner of the eye linked to an infection in the second upper molar, the nasal ala associated with an upper canine infection, and the neck connected to a lower molar infection [1,14].

Studies focusing on the prevalence of sinus tracts have consistently highlighted the relatively rare occurrence of their cutaneous manifestation. Numerous researchers have emphasized the critical need for collaboration between physicians and dentists to prevent patients from undergoing unnecessary biopsies, prolonged antibiotic treatments, or avoidable surgeries prior to establishing an accurate diagnosis and initiating appropriate endodontic therapy. Cutaneous sinus tracts are typically a consequence of underlying pathology, and it is essential for clinicians to identify the root cause effectively [9,15].

The fascial planes of the head and neck, first thoroughly documented by Grodinsky and Holyoke in 1938, serve as pathways of least resistance, allowing the spread of orofacial infections [16]. Pathogenic microorganisms produce enzymes such as hyaluronidase and collagenase, which break down surrounding tissues and facilitate the infection’s progression through these spaces. In this particular case, the perforation of the buccal cortical plate is an essential factor that enables the infection to invade the fascial spaces [17].

In the described case, the extraoral drainage of infection associated with the mandibular right second molar likely occurred because the infection spread beneath the attachment of the depressor anguli oris muscle. For mandibular teeth, dental abscesses can drain extraorally when the infection extends below the attachments of muscles such as the mylohyoid or buccinator. In contrast, for maxillary teeth, the extraoral drainage of a dental abscess occurs when the infection progresses above the level of the buccinator muscle attachment.

Odontogenic cutaneous lesions involve a draining sinus fistula originating from pus accumulation and granulation tissue in the alveolar bone. While most odontogenic fistulas drain intraorally, chronic dental infections can lead to gradual bone destruction, forming an abscess. Once the infection penetrates the cortical bone and periosteum, it extends into the surrounding soft tissues, where its spread is limited by muscle attachments and facial planes [5].

The variable presentation and location of odontogenic cutaneous sinus fistulas often lead to diagnostic confusion. These fistulas can manifest on the skin as dimples, nodules, abscesses, cysts, ulcers, draining lesions, or nodulocystic formations with suppuration. While their position typically correlates with the affected teeth, the nonspecific nature of these skin changes complicates diagnosis. In the presented case, we observed a transformation in the skin lesion's appearance, evolving from a bi-lobed mass to a slightly larger nodule, accompanied by a significant change in fistula activity, progressing from an inactive crust to a lateral orifice discharging serous fluid. Furthermore, misdiagnosis has been observed in edentulous patients, where infections involving implants, bones, or bone grafts are the underlying cause [5,9].

The cutaneous presentation of odontogenic sinuses typically consists of small, symmetrical, erythematous, and non-tender nodulocystic lesions. On physical examination, palpable tissue cords often connect the skin to the underlying maxilla or mandible. Compression of these cords may result in the expulsion of a pus-like discharge through a small central opening, or punctum, in the sinus. Perilesional skin may exhibit slight retraction, creating a dimpling effect. In this case, the initial presentation of the cutaneous lesion consisted of two small nodules, which later merged into a single large nodule with a draining fistula.

Additional examinations primarily involve radiographic imaging, which is essential to identify the chronic periradicular infectious focus. Panoramic X-rays and periapical dental radiographs play a crucial role in detecting cutaneous odontogenic sinuses, as they can reveal radiolucent regions around the affected teeth. However, these diagnostic tools are typically employed only when a dental origin is suspected. In cases where the diagnosis remains unclear, cone beam computed tomography (CBCT) imaging can offer invaluable information about the source and extent of the infection. CBCT scans may highlight bony changes, such as thinning or disruption of the alveolar cortical plate near the affected tooth, soft tissue abnormalities, including muscle thickening and loss of surrounding fat layers, and alterations in the paranasal sinuses, such as mucosal thickening, fluid accumulation, and thickened bony structures [4,6]. Gutta-percha point is typically inserted into the fistula's opening before performing an X-ray, allowing the visualization of the fistulous tract's pathway and identifying its source [1,9].

When evaluating chronic draining sinus tracts on the face or neck, dental infections should be considered a leading cause. Although conditions such as carcinomas, furuncles, osteomyelitis, bacterial infections, congenital fistulas, and pyogenic granulomas can also give rise to cutaneous sinus tracts, dental origins are often the primary etiology in such cases [18]. In this case, the initial diagnosis of the cutaneous lesion suggested a dental etiology, but the possibility of a systemic infection led us to prescribe serological tests to ensure a responsible approach.

The treatment of odontogenic cutaneous sinus tracts focuses on eliminating the underlying dental infection. Non-surgical root canal therapy is the preferred approach when the affected tooth is salvageable, as it removes the infected pulp and promotes healing. Some authors advocate for surgical excision of the fistulous tract due to the erroneous belief that it is lined with epithelium. In the case presented, the tooth was deemed non-restorable and was therefore extracted. This procedure was followed by the complete removal of the cord, extending from its origin to its attachment at the skin. This approach resulted in the relaxation of the facial skin, and the restoration of normal facial contours. Adjunctive antibiotic therapy may be used to manage acute inflammation and support healing, but it is not a substitute for definitive dental treatment. Once the source is addressed, the sinus tract typically resolves spontaneously without requiring specific skin intervention. However, in persistent cases with residual scarring, minor dermatological procedures can be considered for cosmetic purposes. Regular follow-up with radiographic imaging is essential to confirm the resolution of the infection and ensure proper healing, preventing recurrence [9,19].

This case report is unique because it highlights an uncommon presentation of an odontogenic cutaneous sinus tract, a condition that is often misdiagnosed due to its uncommon nature and atypical manifestation. Such misdiagnoses can delay appropriate dental treatment, as these tracts are frequently mistaken for dermatological conditions or other non-odontogenic pathologies. By documenting this case, we aim to raise awareness among clinicians, particularly non-dental professionals, about the importance of considering dental origins in cases of unexplained cutaneous lesions.

The take-home lesson is the critical need for a thorough clinical and radiographic examination when evaluating chronic cutaneous lesions in the facial or cervical region. Timely diagnosis and management of the underlying odontogenic infection can lead to resolution without unnecessary interventions.

## Conclusions

Odontogenic cutaneous sinus tracts, though rare, pose significant diagnostic and therapeutic challenges. Misdiagnosis often results in unnecessary interventions, delaying appropriate treatment. Accurate diagnosis requires a thorough clinical and radiographic assessment. Timely and targeted management is essential to resolve these benign but impactful conditions effectively. Raising awareness among healthcare providers about the characteristics and management of these lesions is essential to reduce misdiagnosis and improve patient care.
